# Synthesis of Chlorine- and Nitrogen-Containing Carbon Nanofibers for Water Purification from Chloroaromatic Compounds

**DOI:** 10.3390/ma15238414

**Published:** 2022-11-25

**Authors:** Anna M. Ozerova, Arina R. Potylitsyna, Yury I. Bauman, Elena S. Tayban, Inna L. Lipatnikova, Anna V. Nartova, Aleksey A. Vedyagin, Ilya V. Mishakov, Yury V. Shubin, Olga V. Netskina

**Affiliations:** 1 Boreskov Institute of Catalysis SB RAS, Lavrentieva Av. 5, 630090 Novosibirsk, Russia; 2 Department of Natural Sciences, Novosibirsk State University, Pirogova Str. 2, 630090 Novosibirsk, Russia; 3 Nikolaev Institute of Inorganic Chemistry SB RAS, Lavrentieva Av. 3, 630090 Novosibirsk, Russia

**Keywords:** chloroaromatics, adsorption, hydrodechlorination, N-containing carbon nanofibers, trichloroethylene, nickel catalyst

## Abstract

Chlorine- and nitrogen-containing carbon nanofibers (CNFs) were obtained by combined catalytic pyrolysis of trichloroethylene (C_2_HCl_3_) and acetonitrile (CH_3_CN). Their efficiency in the adsorption of 1,2-dichlorobenzene (1,2-*DCB*) from water has been studied. The synthesis of CNFs was carried out over self-dispersing nickel catalyst at 600 °C. The produced CNFs possess a well-defined segmented structure, high specific surface area (~300 m^2^/g) and high porosity (0.5–0.7 cm^3^/g). The addition of CH_3_CN into the reaction mixture allows the introduction of nitrogen into the CNF structure and increases the volume of mesopores. As a result, the capacity of CNF towards adsorption of 1,2-*DCB* from its aqueous solution increased from 0.41 to 0.57 cm^3^/g. Regardless of the presence of N, the CNF samples exhibited a degree of 1,2-*DCB* adsorption from water–organic emulsion exceeding 90%. The adsorption process was shown to be well described by the Dubinin–Astakhov equation. The regeneration of the used CNF adsorbent through liquid-phase hydrodechlorination was also investigated. For this purpose, Pd nanoparticles (1.5 wt%) were deposited on the CNF surface to form the adsorbent with catalytic function. The presence of palladium was found to have a slight effect on the adsorption capacity of CNF. Further regeneration of the adsorbent-catalyst via hydrodechlorination of adsorbed 1,2-*DCB* was completed within 1 h with 100% conversion. The repeated use of regenerated adsorbent-catalysts for purification of solutions after the first cycle of adsorption ensures almost complete removal of 1,2-*DCB*.

## 1. Introduction

Chlorinated organic compounds (COCs) are among the most persistent and toxic pollutants. They are widely used as pesticides, propellants, refrigerants, degreasers, solvents, as well as reagents and intermediate products in organic synthesis [1,2]. The use of COCs in various technological processes is accompanied by their release into the environment. On the other hand, the chlorination of water-containing organic substances may also result in the formation of a number of COCs which have carcinogenic and mutagenic effects [3]. COCs are known to be very stable and only slightly dissolvable. They have a high resistance to degradation. Considerable amounts of COCs can be found in industrial effluents, groundwater, rivers, seas, rainwater, and even in drinking water [1,2,4]. Therefore, the research aiming to improve the purification technologies for removing such dangerous chemicals from liquid waste and wastewater is of high demand. Various methods have been proposed for the removal of COCs from water, including catalytic oxidation [5]; biodegradation [6]; advanced oxidation processes, such as photocatalytic destruction [7] or electrochemical degradation [8]; reductive dechlorination [9]; as well as non-destructive methods, such as adsorption [10,11,12]. Each of these methods has its advantages and limitations [1]. For example, catalytic oxidation technology has been widely utilized because of its relative simplicity. Meanwhile, it remains costly, requires elevated temperatures, and poses a certain risk to the environment due to the potential emission of highly toxic byproducts (e.g., dioxins, furans, and phosgene) derived as a result of their incomplete combustion [13].

The catalytic pyrolysis of chlorinated hydrocarbons and related wastes is known to be one of the most powerful techniques for their processing [14,15]. This process is carried out in a reductive atmosphere, which completely eliminates the risk of the formation of side ecotoxicants. The self-dispersing Ni-based catalysts can be used for this purpose as the most active and tolerant to deactivation by chlorine systems [16]. “Self-dispersing” implies fast fragmentation (disintegration) of bulk Ni or Ni-based alloys (Ni-M, where M = Pd, Mo, W etc.) in the reaction atmosphere, which leads to the formation of disperse active particles [17]. The decomposition of aliphatic chlorinated hydrocarbons (i.e., 1,2-dichloroethane (DCE) and trichloroethylene (TCE)) over alloyed Ni-M catalysts is accompanied by accumulation of the chlorine-containing graphite-like nanomaterials represented mostly by carbon nanofibers (CNFs) [17,18,19,20]. The filamentous carbon product resulted from the catalytic decomposition of chlorinated hydrocarbons is known to possess both a unique segmental structure and high textural parameters (specific surface area of 300–400 m^2^/g, pore volume of 0.5–0.8 cm^3^/g), which makes this type of nanomaterial very attractive for adsorption applications [21,22]. The appearance of the segmented secondary structure of CNFs is believed to be driven by the periodic ‘chlorination-dechlorination’ process taking place over the surface of nickel catalyst during the H_2_-assisted catalytic pyrolysis of chlorinated hydrocarbons [23]. Thus, the potential use of such carbon nanomaterial obtained via catalytic pyrolysis of COCs is of particular practical interest.

It should be noted that real industrial organochlorine wastes are usually composed of a complex mixture of polychlorinated hydrocarbons, including a portion of organics functionalized by O- and N-containing groups. As recently demonstrated, the use the nitrogen-containing compound acetonitrile (AN) as a co-reagent in the catalytic pyrolysis of DCE or TCE vapors allows the obtainment of N-containing CNF materials with a nitrogen content of up to 3 wt% [24]. The addition of AN vapors into the reaction mixture was also shown to have a boosting effect on the catalyst’s productivity with respect to the CNF material. In addition, the produced N-containing CNF (N-CNF) exhibited the preserved segmental structure of fibers, along with an enhanced specific surface area (up to 550 m^2^/g) and porosity [24]. The high textural parameters of such filamentous carbon product make it a very attractive nanomaterial for various fields of application, including adsorption [25,26].

Carbon materials are commonly used as adsorbents for water purification from diverse types of contamination [27,28]. As recently shown, CNFs produced via the catalytic decomposition of DCE demonstrate very high efficiency in adsorption of chloroaromatic pollutants from water [29,30]. At the same time, the possibility of reusing the carbon adsorbent for water purification should include the removal of the chloroaromatic compound adsorbed during the first purification cycle. Thus, the regeneration of the adsorbent can be carried out under mild conditions using the catalytic reaction of liquid-phase hydrodechlorination (HDC) [22,31,32]. HDC is a widespread method that is recognized as the most effective method for the degradation of polychlorinated aromatics. Various supported catalysts containing Pd, Pt, Ni and its alloys can be used as active components for this purpose [33,34]. In the case of using carbon adsorbents, palladium at a moderate concentration of 1–3% is to be deposited on their surface in order to provide the necessary catalytic function [35]. In other words, the purification cycle involves the adsorption of COCs from the aqueous solution and the reductive regeneration of the catalyst by the liquid-phase HDC of adsorbed species with molecular hydrogen [21]. Such an approach allows for both concentrating the chloroaromatic pollutants and obtaining the products useful in the chemical industry. The object of study for water purification was 1,2-dichlorobenzene (1,2-*DCB*), which is a high-priority pollutant. It is widely used as a high-boiling solvent in chemical industry, as well as a reagent and an intermediate for dyes and agrochemical manufacture [36].

In the present paper, a complex approach for the disposal of the chlorinated organic multi-component wastes is proposed. In the first stage, TCE, which was chosen as the model aliphatic polychlorinated hydrocarbon, was converted over Ni-catalyst into segmented CNF material. In addition, the N-containing CNF was obtained in the same way via combined pyrolysis of TCE and AN. In the second stage, the produced CNF and N-CNF samples were explored in the adsorption of 1,2-*DCB* from aqueous solutions in the static regime. The opportunity to regenerate the spent CNF adsorbent by liquid-phase catalytic hydrodechlorination was also examined. For this purpose, 1.5 wt% palladium was deposited on both of the CNF samples. These adsorbent-catalysts were then tested in the adsorption–regeneration cycle.

## 2. Materials and Methods

**Figure 1:**
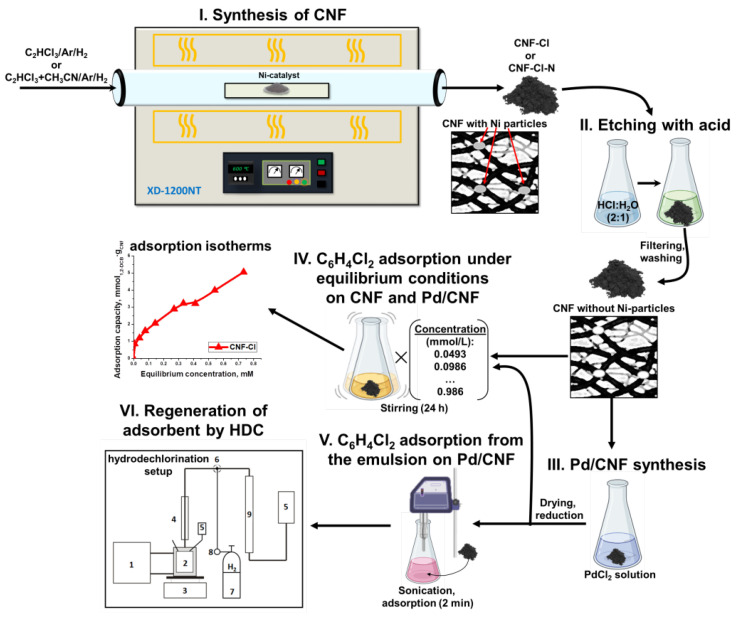
Schematic representation of the experimental work. The HDC setup consists of the following parts: (1) thermostat; (2) temperature-controlled reactor; (3) magnetic stirrer; (4) reflux condenser; (5) odor trap; (6) three-way valve; (7) gas bottle; (8) gas reducer; (9) measuring burette.

A schematic representation of the principle experimental stages is presented in Figure 1. A detailed description of each step is given below.

The following commercial reagents were used as received: trichloroethylene (C_2_HCl_3_, chemically pure, Component-Reactiv, Moscow, Russia); acetonitrile (CH_3_CN, chemically pure, Component-Reactiv, Moscow, Russia); PdCl_2_ (pure, Aurat, Moscow, Russia); NaBH_4_ (purity of 98 wt%, Chemical Line, Saint Petersburg, Russia); 1,2-dichlorobenzene (C_6_H_4_Cl_2_, purity of 99 wt%, Sigma-Aldrich, St. Louis, MO, USA); KOH (analytically pure, Reakhim, Moscow, Russia); and 2-propanol (CH_3_CH(OH)CH_3_, high purity grade, Baza No.1 Khimreactivov, Staraya Kupavna, Russia). Microdispersed Ni-catalyst was synthesized as described elsewhere [37].

### 2.1. Synthesis of Functionalized CNFs

The H_2_-assisted catalytic pyrolysis of C_2_HCl_3_ vapors (or the joint decomposition of C_2_HCl_3_ and CH_3_CN) resulting in the formation of carbon nanomaterial was carried out in a flow-through horizontal quartz reactor (Zhengzhou Brother Furnace Co., Ltd., Zhengzhou, China).

A specimen of the catalyst (microdispersed powder of Ni), 300.00 ± 0.5 mg each, was spread throughout the entire quartz plate. The plate was then placed inside the reactor, after which the reactor was heated in an Ar atmosphere up to the reaction temperature of 600 °C. The samples were then treated in a hydrogen flow at 600 °C for 10 min in order to reduce the oxide film on their surfaces. After the reduction, the reactor was fed with the reaction mixture containing vapors of TCE (6 vol%) or TCE (6 vol%) + AN (25 vol%), hydrogen (37 vol%), and argon, at 600 °C. The flow rate of the reaction mixture was 54 L/h. The duration of the synthesis was 2 h. When the experiment was completed, the reactor was cooled in an argon flow to room temperature, after which the carbon product was unloaded and weighed. The measured carbon yield, Y_C_ (expressed in grams of CNF per 1 g of catalyst, g_CNF_·g_cat_^−1^), was found to be 9.8 g_CNF_·g_cat_^−1^ (for TCE only) and 10.6 g_CNF_·g_cat_^−1^ (in the case of TCE + AN).

The resulting carbon nanomaterials were further treated with hydrochloric acid (12%) for 12 h in order to remove the metallic particles of residual catalyst. The etched CNF material was then washed with water to neutral pH, filtered out, and dried at 120 °C. The resulting CNF material produced via the decomposition of TCE only was labeled as «CNF-Cl», whereas the CNF sample obtained by the joint decomposition of TCE and AN was labeled as «CNF-Cl-N».

### 2.2. Synthesis of the Pd/CNF Adsorbent-Catalysts

The adsorbent-catalysts were prepared by incipient wetness impregnation of the CNF-Cl and CNF-Cl-N samples with hydrochloric solutions of PdCl_2_. The obtained samples were dried at 110–130 °C for 4 h. The reduction of deposited PdCl_2_ was performed at room temperature by treatment with an aqueous solution of NaBH_4_ (Pd:NaBH_4_ molar ratio was 1:3). The synthesized adsorbent-catalysts were labeled as «Pd/CNF-Cl» and «Pd/CNF-Cl-N». According to X-ray fluorescence analysis data, the palladium content in all the Pd-containing samples was 1.5 ± 0.05 wt%.

### 2.3. Study on 1,2-DCB Adsorption from Aqueous Solution under Equilibrium Conditions

#### 2.3.1. Measurement Procedure

The adsorption characteristics of the prepared CNF and Pd/CNF samples were studied under equilibrium conditions by a static method at 25 °C for 24 h. For this purpose, 3 mg of each sample was added to a 60 mL solution of 1,2-*DCB*, with concentrations in the range from 0.0493 to 0.986 mmol·L^−1^. In order to exclude the effect of the pore-diffusion factor, the mixtures were evenly shaken with a shaker LOIP LS-110 (RNPO RusPribor, Saint-Petersburg, Russia) at an orbiting speed of 200 rpm. Changes in the concentration of 1,2-*DCB* during the adsorption cycle were monitored by the UV-spectroscopy technique [38,39,40] using a Varian Cary 100 instrument (Agilent, Santa Clara, CA, USA). To obtain reliable data, each experiment was repeated at least three times. Differences between experimental values did not exceed ±3%.

The amount of the adsorbed 1,2-*DCB* was calculated as follows [41]:

(1) $$A=\frac{\Delta C\times V}{m}$$

where *A* is the adsorption capacity of the CNFs or Pd/CNF, mol·g^−1^; Δ*C* is the difference in 1,2-*DCB* concentration before and after adsorption, mol·L^−1^; *V* is the volume of 1,2-*DCB* solution, L; *m* is the loading of the adsorbent, g.

The pore filling was determined as a ratio of the volume of 1,2-*DCB* in CNF pores to the total pore volume:

(2) $$F_{pores}=\frac{A^{*}}{V_{\sum pore}}\times 100\%$$

where *F_pores_* is the pore filling of 1,2-*DCB*, %; *A*^∗^ is the adsorption capacity, which was achieved by adsorption of 1,2-*DCB* from an aqueous solution, cm^3^·g^−1^; *V*_Σ_*_pore_* is the total pore volume of CNF, cm^3^·g^−1^.

#### 2.3.2. Adsorption Isotherm Modeling

Both the Langmuir and Dubinin–Astakhov isotherm models were used to analyze the adsorption data. The applicability of the models was checked via a comparison of their correlation coefficients (R^2^ values).

##### Langmuir Isotherm

The Langmuir isotherm is often used to study the adsorption processes of various pollutants. This model is applicable for monolayer adsorption onto a surface with a limited amount of identical sites. Uniform adsorption energies and no adsorbate transmigration in the plane of the surface are considered. The equation for the Langmuir isotherm is given below [42]:

(3) $$A=\frac{K_{L}A_{\max}C_{eq}}{1+K_{L}C_{eq}},$$

where A is the amount of 1,2-*DCB* adsorbed per gram, mol·g^−1^; $A_{\max}$ is the adsorption capacity or the adsorption maximum, mol·g^−1^; $C_{eq}^{}$ is the equilibrium 1,2-*DCB* concentration, mol·L^−1^; $K_{L}$ is the Langmuir constant, L·mol^−1^.

The surface area of adsorbent occupied by one molecule of 1,2-*DCB* (ω) was calculated by the Langmuir isotherm model [43]:

(4) $$\omega =\frac{SSA}{A_{\max}N_{A}}$$

where SSA is the specific surface area of the adsorbent, m^2^·g^−1^; $A_{\max}$ is the adsorption capacity or the adsorption maximum, mol·g^−1^; $N_{A}$ is the Avogadro number (6.02 × 10^23^ mol^−1^).

##### Dubinin-Astakhov Isotherm

As is reported [44], the Dubinin theory, which considers volume filling for vapor adsorption, can be applied to study the adsorption of organic compounds from their solutions [45,46]. The Dubinin–Astakhov equation can be expressed as follows [47]:

(5) $$A=A_{\max}\exp\left[-{\left[\frac{RT\times \ln(\frac{C_{\max}}{C_{eq}})}{E_{ads}^{eff}}\right]}^{n}\right],$$

where $A_{\max}$ is the adsorption maximum or adsorption capacity, mol·g^−1^; $C_{\max}^{}$ is the highest concentration of 1,2-*DCB* in water, mol·L^−1^; $C_{eq}^{}$ is the equilibrium 1,2-*DCB* concentration, mol·L^−1^; $E_{ads}^{eff}$ is the characteristic energy of 1,2-*DCB* adsorption, kJ·mol^−1^.

The maximum amount of 1,2-*DCB* (*W*_0_) adsorbed per gram (or maximum adsorption capacity), and the average diameter (*d*) of the adsorbent’s pores filled with 1,2-*DCB* were defined by the Dubinin–Astakhov isotherm model [21,43]:

(6) $$W_{0}\;=\;\frac{A_{\max}\;\times \;M_{1,2-DCB}}{\rho_{1,2-DCB}},$$

(7) $$d=2\cdot \frac{K_{C_{6}H_{6}}\times \beta }{E_{ads}^{eff}},$$

where $M_{1,2-DCB}$ is the molar mass of 1,2-*DCB*, g·mol^−1^; $\rho_{1,2-DCB}$ is the density of 1,2-*DCB*, g·cm^−3^; $K_{C_{6}H_{6}}$ is the coefficient for the standard (benzene), equal to 12 kJ·nm·mol^−1^; *β* is the affinity coefficient (for 1,2-*DCB*, equal to 1.28).

### 2.4. Testing Pd/CNF in the Adsorptive-Catalytic Cycle

Purification of the aqueous medium from 1,2-*DCB* was conducted in two steps comprising an adsorptive–catalytic cycle: (1) adsorption of 1,2-*DCB* on Pd/CNF-Cl and Pd/CNF-Cl-N samples from the emulsion; (2) regeneration of adsorbent-catalysts by hydrodechlorination in a liquid phase. First, an emulsion of 0.15 mL of 1,2-*DCB* in 100 mL of water was prepared by sonication for 20 min, after which 0.5 g of catalyst was added. The adsorption time was 2 min while shaking. The catalyst was then separated by decantation (2 min) and immediately used in the liquid-phase hydrodechlorination experiment.

The calculation of the maximum amount of 1,2-*DCB* in the reaction medium during the regeneration of adsorbent-catalysts by hydrodechlorination was performed according to the equation:

(8) $$C_{1,2-DCB}^{\max}=\frac{A\times m\times V_{0}}{V},$$

where $C_{1,2-DCB}^{\max}$ is the concentration of 1,2-*DCB* adsorbed on the adsorbent-catalyst, mmol·L^−1^ (or M); *A* is the adsorption capacity, which was achieved by the adsorption of 1,2-*DCB* from an aqueous emulsion, mol·g^−1^; m is the mass of the adsorbent-catalyst, g; *V*_0_ is the standard volume, 1000 mL; *V* is the volume of 2-propanol in the reaction medium of 1,2-*DCB* hydrodechlorination, 11 mL.

The apparatus for 1,2-*DCB* hydrodechlorination consisted of two main parts: a 50 mL temperature-controlled glass reactor equipped with a magnetic stirrer and a volumetric block to maintain a constant H_2_ pressure. The reaction medium consisted of two immiscible fluids: 11 mL of 2-propanol and 4 mL of a 50 wt% solution of KOH. Alkali was used to bind HCl formed during the hydrodechlorination, thus preventing the catalyst from deactivation [48]. 2-Propanol serves as the organic phase which is able to dissolve 1,2-*DCB* efficiently and facilitate the formation of active hydrogen species on the surface of the catalyst during the hydrodechlorination process [49].

The regeneration of the adsorbent-catalyst by liquid-phase hydrodechlorination of 1,2-*DCB* was performed at 25 °C under a constant hydrogen pressure of 0.1 MPa and continuous stirring at 1200 rpm. When the stirring rate is above 800 rpm, the external diffusion processes have a negligible influence [50]. Following the complete conversion of 1,2-*DCB*, the catalyst was separated from the reaction medium by filtration, washed with water, dried at 110–130 °C for 4 h, and then used in the adsorptive–catalytic cycle for the second run. It is important to note that each experiment was repeated at least 3 times. Differences between the experimental points from different repetitions do not exceed ±4%.

Analysis of the hydrodechlorination products was performed using a Chromos GH-1000 gas chromatograph (Chromos Engineering, Dzerzhinsk, Russia) with a flame-ionization detector and a column (2.5 mm × 2 m) filled with Chromaton N-AW sorbent. The instrument was operated under argon using a hydrogen flame at temperatures from 60 to 120 °C (heating rate 10 °C/min).

### 2.5. Characterization of the Samples

Pure CNF as Pd-containing samples were explored by means of high-resolution transmission electron microscopy (HR TEM) using a JEM-2010CX microscope (JEOL, Tokyo, Japan). The device works at an accelerating voltage of 100 kV. The spherical aberration coefficient of an objective lens is 2.8 mm. The line resolution of the microscope is 1.4 Å.

The X-ray diffraction (XRD) study of the synthesized CNF samples was performed on a Shimadzu XRD-7000 (Shimadzu, Tokyo, Japan) diffractometer (CuK_α_ radiation) at room temperature using a graphite monochromator. The average crystallite size was determined from the integral broadening of the profiles of the 002 diffraction peak described by the Pearson function VII (PVII), using the Scherrer formula in WINFIT 1.2.1 software [51].

X-ray photoelectron spectroscopy (XPS) data were collected on a SPECS (SPECS Surface Nano Analysis GmbH, Berlin, Germany) spectrometer equipped with a hemispherical PHOIBOS-150-MCD-9 analyzer. The non-monochromatic Mg K_α_ radiation (hν = 1253.6 eV) at 180 W was used as the primary excitation. For calibration of the spectrometer, the Au 4f_7/2_ (binding energy (BE) at 84.0 eV) and Cu 2p_3/2_ (932.7 eV) peaks from metallic gold and copper foils were used [52]. The samples were located on a holder with a 3 M double-sided adhesive copper conducting tape. The Peak 4.1 XPS software package was applied for both spectral analysis and data processing. To determine the binding energy values and the areas of XPS peaks, the Shirley background was subtracted, and an analysis of line shapes was performed. Gaussian–Lorentzian functions were used to fit the curves in each XPS area. The atomic ratios of elements were determined from the integral photoelectron peak intensities. The corresponding relative atomic sensitivity factors [52] and the transmission function of the analyzer were used for the correction.

In order to determine the specific surface area (*SSA*) and pore volume (*V_pore_*) of the obtained CNF, nitrogen adsorption/desorption at 77 K (Brunauer–Emmett–Teller (BET) method) was used. The samples were degassed under an oil-free vacuum at 300 °C for 5 h. The isotherms of adsorption were measured using an ASAP-2400 automated instrument (Micromeritics, Norcross, GA, USA).

The loading of palladium in adsorbent-catalysts was measured by means of X-ray fluorescence analysis using a VRA-30 instrument (Carl Zeiss, Jena, Germany) with a Cr anode X-ray tube. The relative determination error was ±5%.

## 3. Results and Discussion

### 3.1. Characterization of CNF Samples

**Figure 2:**
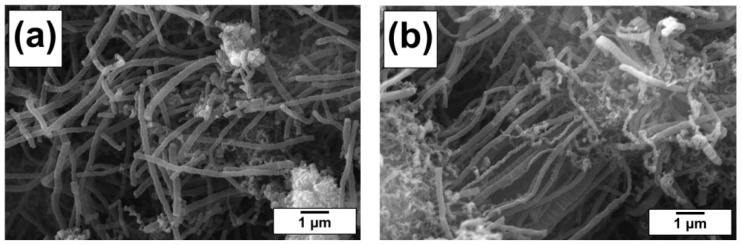
SEM micrographs of the CNF-Cl (**a**) and CNF-Cl-N (**b**) samples.

The morphology and structure of the synthesized carbon nanomaterial was studied by scanning and transmission electron microscopies. According to the SEM data, the carbon products formed as a result of the catalytic decomposition of TCE and TCE + AN mixtures over Ni catalyst are predominantly represented by rather long graphite-like filaments (Figure 2). Note that the addition of AN to the reaction mixture has no evident effect on the morphology of the produced carbon nanomaterial.

**Figure 3:**
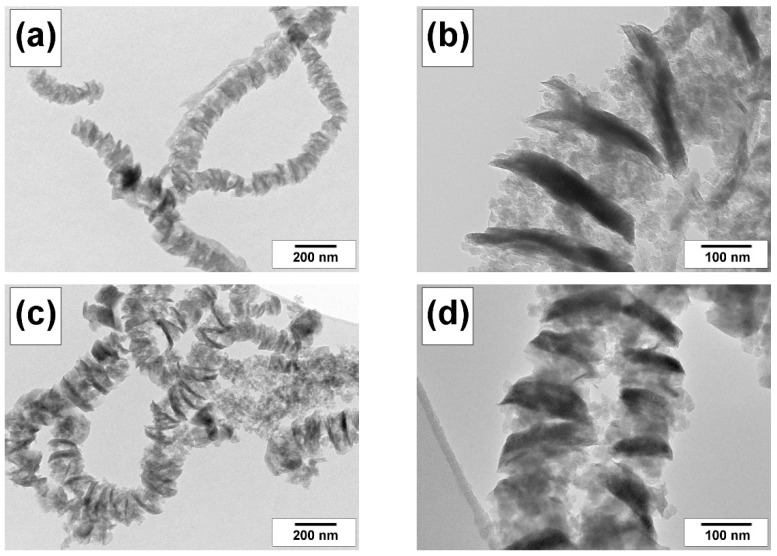
TEM micrograph of (**a**,**b**) CNF-Cl and (**c**,**d**) CNF-Cl-N.

The detailed structure of CNF samples is represented by the selected TEM images shown in Figure 3. It is also clear from TEM data that the addition of AN into the TCE/H_2_/Ar reaction mixture (Figure 3c,d) does not have any noticeable impact on the structure of segmented carbon filaments. The formation of such a structure is caused by the periodic process of chlorination–dechlorination, which takes place on the surface of active Ni particles, catalyzing the growth of CNF. The dark sections of the fibers are densely packed graphite-like “flakes”, while the lighter ones are represented by graphite packs with a looser arrangement and a greater interplanar spacing [23]. The diameter of the carbon fibers varies from 100 to 300 nm.

**Figure 4:**
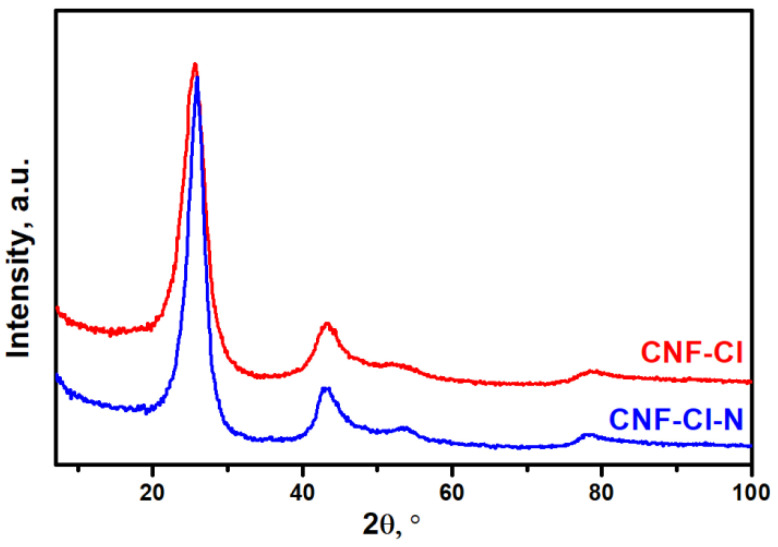
XRD patterns for the synthesized CNF-Cl and CNF-Cl-N samples. Both samples were washed from metallic particles by etching with HCl acid.

XRD patterns of the synthesized CNF materials exposed to acidic treatment are presented in Figure 4. Both the CNF-Cl and CNF-Cl-N samples showed a broad reflection from the (002) graphite planes at a 2θ of 25–26° (Figure 4). No reflexes attributed to traces of the metallic component (Ni) were identified. The distance between the graphitic layers (d_002_) determined from the position of the (002) peak was found to be 3.50 Å and 3.45 Å for CNF-Cl and CNF-Cl-N, respectively. It should be noted that the calculated average distance between the layers in CNFs (3.45–3.50 Å) is significantly larger than that corresponding to the crystalline graphite (3.35 Å).

**Figure 5:**
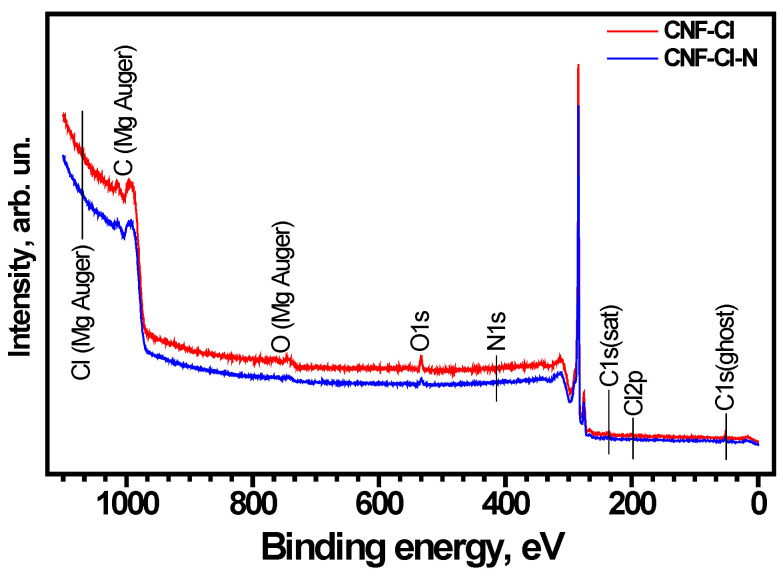
Survey XPS spectra of the CNF-Cl and CNF-Cl-N samples.

**Figure 6:**
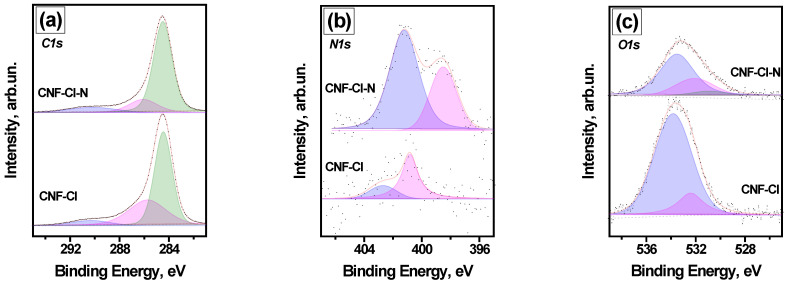
(**a**) C1s, (**b**) N1s, (**c**) O1s XPS spectra of the CNF-Cl and CNF-Cl-N samples.

The functional groups present on the surface of the obtained CNF samples were studied using the XPS method (Tables S1 and S2). As seen in Figure 5, the main elements are carbon and oxygen. In addition to the corresponding lines, the detailed analysis of survey XPS spectra pointed to the presence of N1s and Cl2p low-intensity lines as well. After survey recording, the region spectra for the main lines of elements were collected to obtain a sufficient ‘signal/noise’ ratio for peak deconvolution and to define the positions of the peaks and analyze their area (Figure 6). The calculated contents of the elements (in atomic percent) are presented in Table 1. The results of the detailed quantitative XPS data analysis are presented in Table 2 and Figure 6.

The oxygen-containing groups appear in the samples as a result of their contact with atmospheric air after their removal from the reactor (see Table 2). The CNF-Cl-N sample contains the greatest amount of functional groups (0.39 wt% of N and 0.17 wt% of Cl). It was found that the introduction of nitrogen into the CNF structure promotes an almost 2-fold increase in the chlorine concentration (from 0.09 to 0.17 wt%). Presumably, the higher content of Cl in the CNF-Cl-N sample might prevent the adsorption of oxygen, thus leading to a drop in O concentration (from 2.65 to 1.47 wt%). It should be also noted that an insignificant amount of nitrogen was also detected in the composition of the CNF-Cl sample (Table 1).

The C1s spectra of the CNF samples are shown in Figure 6a. Since the amounts of O, N, and Cl are low, the presence of the components of C1s spectra attributed to the carbon bound with these elements is not expected. For both CNF-Cl and CNF-Cl-N samples, the C1s line consists of three components: C-C sp^2^ (binding energy at 284.5 eV), C-C (C-H) sp^3^ (at 286 eV), and π→π* shake-up satellite (at 290 eV) [53,54,55]. The comparison of the C1s line shapes shows a drop in the portion of sp^3^ carbon for the CNF-Cl-N sample (C sp^3^ portion of 0.17) when compared with the CNF-Cl sample (C sp^3^ portion of 0.36).

The N1s spectra of the studied samples are presented in Figure 6b. Analysis of the N1s region for the CNF-Cl sample reveals the presence of two components at 400.8 eV, described as N-O bonds [56], and at 402.7 eV, assigned to quaternary N [55], trapped N_2_ [56] or O=N-C groups [57]. For the CNF-Cl-N sample, the components of the N1s line at 398.5 eV attributed to N≡C [56] and at 401.2 eV related to C-(NC)-C graphitic nitrogen in aromatics [57] were found in a ratio of 1:2.

In Figure 6c, the O1s spectra of the studied samples are demonstrated. Peaks were observed in the spectra of the CNF-Cl sample at 532.4 eV (C=O) and 533.8 eV (C-OH), and at 531.0 eV (C(O)O), 532.1 eV (C=O), and 533.6 eV (C-OH) for the CNF-Cl-N sample [58,59]. It should be noted that the total amount of oxygen in the CNF-Cl-N sample is lower if compared with the CNF-Cl sample.

The textural parameters of the obtained carbon nanomaterials were studied by low-temperature adsorption of nitrogen. According to the data obtained (Table 3), the specific surface area of the materials is almost the same: 297 m^2^·g^−1^ for CNF-Cl and 292 m^2^·g^−1^ for CNF-Cl-N. This observation testifies once again to the fact that the addition of AN to the reaction mixture (TCE/H_2_/Ar) does not alter the structural and textural properties of the resulting carbon product.

**Figure 7:**
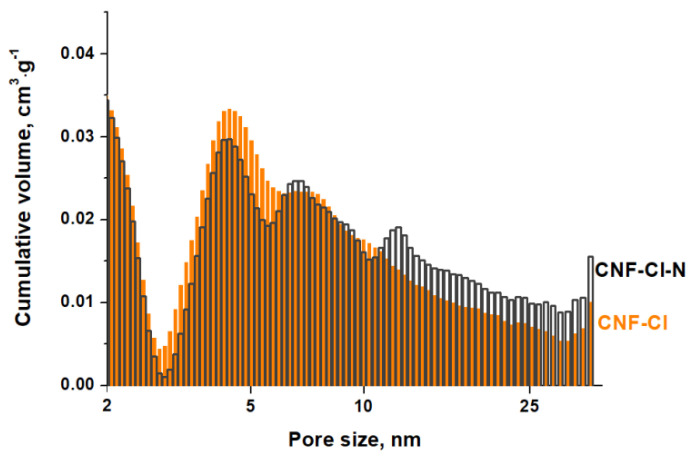
Pore size distribution for the CNF-Cl and CNF-Cl-N samples.

The pore structure of the produced CNF samples was also found to be very similar. Both samples show a bimodal pore size distribution with peaks in micro- and mesoporous regions (Figure 7). The volume of micropores is almost the same and does not exceed 3% of the total pore volume (Table 3). However, the total pore volume and the average pore diameter for the N-containing CNF-Cl-N sample are greater than those for CNF-Cl. The measured values are 0.69 cm^3^·g^−1^ and 9.4 nm for CNF-Cl-N and 0.55 cm^3^·g^−1^ and 7.4 nm for CNF-Cl, respectively (Table 3). The observed difference is due to a larger contribution of mesopores in the case of N-containing CNF (Figure 7).

Thus, the catalytic H_2_-assisted decomposition of TCE (or the mixture of TCE and AN) over Ni-catalyst allowed for the filamentous carbon material to be obtained. The produced carbon nanofibers have a diameter of 100–300 nm and a well-defined segmented structure of densely and loosely packed Cl-containing graphite layers. They are characterized by a comparatively high value of *SSA* (~300 m^2^·g^−1^) and a bimodal pore size distribution. The use of AN as a co-reagent for CNF synthesis results in the incorporation of nitrogen atoms into the structure of carbon product (0.39 wt%), along with an enhancement of total porosity (from 0.55 to 0.69 cm^3^·g^−1^), owing to the increased value of mesopores.

**Figure 8:**
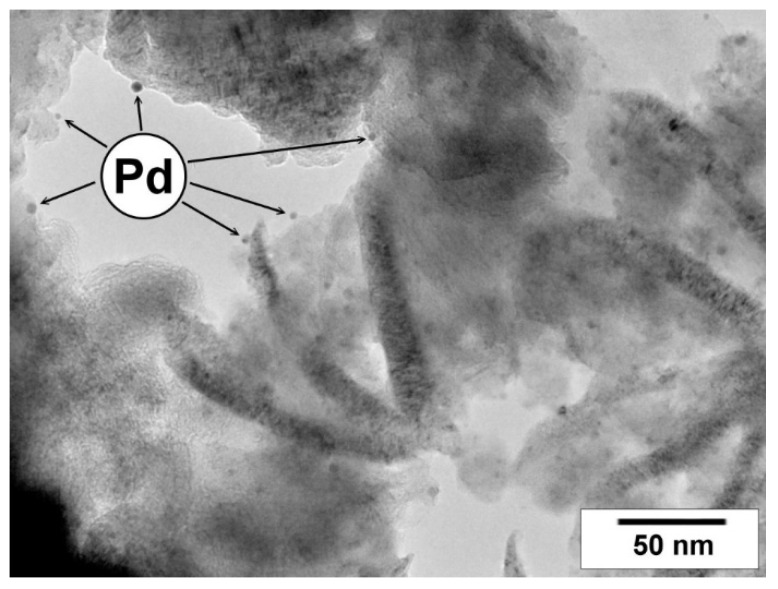
TEM micrograph of the Pd/CNF-Cl-N sample with spherical Pd particles.

Using the obtained CNFs, the palladium adsorbent-catalysts with a Pd content of 1.50 ± 0.03 wt% were prepared. When palladium is supported on CNF, the specific surface area decreases from 297 to 293 m^2^·g^−1^ and from 292 to 284 m^2^·g^−1^ for CNF-Cl and CNF-Cl-N, respectively. Such insignificant changes are due to the localization of palladium nanoparticles on the outer surface of carbon fibers (Figure 8). Pd does not enter their pores, as in the case of microporous carbon black [60].

### 3.2. Adsorbtion of 1,2-DCB from Aqueous Solutions

**Figure 9:**
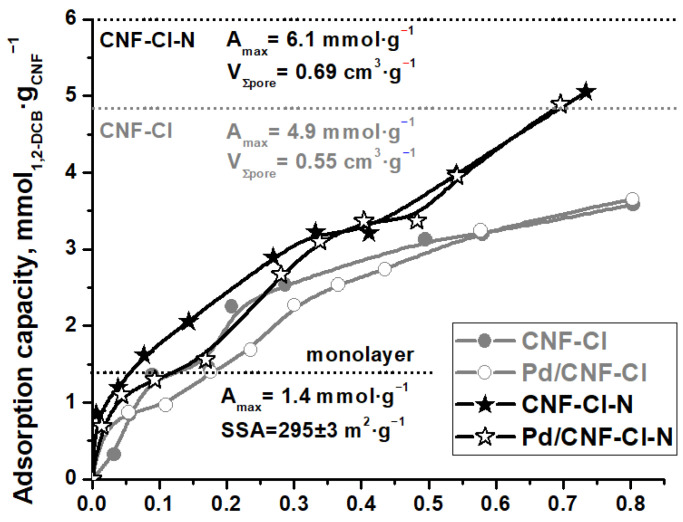
Experimental adsorption isotherms of 1,2-*DCB* at 25 °C on the CNF-Cl, Pd/CNF-Cl, CNF-Cl-N, and Pd/CNF-Cl-N samples.

To characterize the adsorption properties of the synthesized CNF samples and related Pd/CNF materials, the 1,2-*DCB* adsorption from aqueous solutions under equilibrium conditions at 25 °C was investigated. The experimental adsorption isotherms (Figure 9) were analyzed using both the Langmuir and Dubinin–Astakhov models (Table 4).

**Figure 10:**
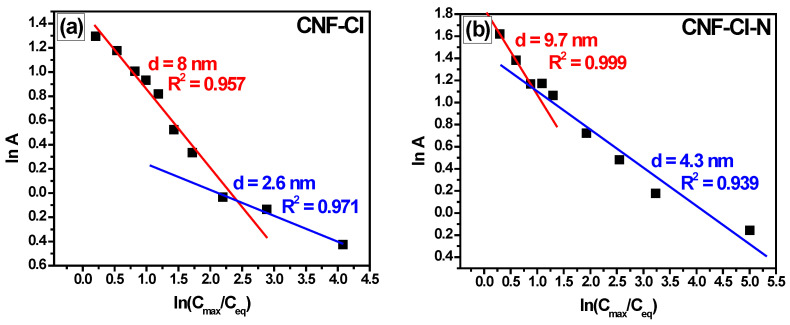

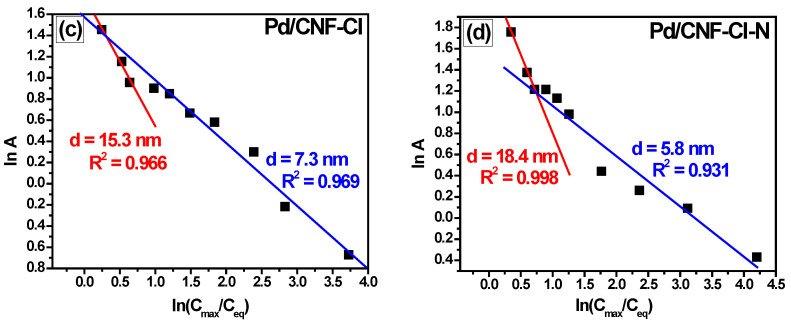
Dubinin–Astakhov model fitting results with *n* = 1 for 1,2-*DCB* adsorption on (**a**) CNF-Cl, (**b**) CNF-Cl-N, (**c**) Pd/CNF-Cl, and (**d**) Pd/CNF-Cl-N samples at 25 °C.

The obtained adsorption isotherms are presented in Figure 9. They are characterized by an inflection in a low range of 1,2-*DCB* concentration (Figure 9), which corresponds to a monolayer capacity of 0.0014 mol·g^−1^, calculated considering the *SSA* value of these carbon materials (Table 4), and a 1,2-*DCB* molecule area of 0.35 nm^2^ [61]. As the 1,2-*DCB* concentration increases, the adsorption capacity of CNF increases as well. This is most probably due to the volumetric filing of pores, as reported recently [21,33]. Indeed, the experimental isotherms are not linearized by the Langmuir Equation (2), but well described by the Dubinin–Astakhov Equation (4) (Table 4), which has been proposed for the mathematical description of the volumetric filling of pores [62]. The empirical parameter *n*, varying from 1 to 6, is linked to the degree of heterogeneity of the pore system. In our case, *n* is equal to 1. It is consistent with the literature that *n* approaches one for adsorbents with a wide pore distribution, including those with a bimodal structure [63]. As can be seen from the fitting results presented in Figure 9, the Dubinin–Astakhov model with *n* = 1 describes the adsorption of 1,2-*DCB* on CNF samples well, which are the bimodal adsorbents with two types of pores (Figure 7). The average diameter of the pores filled with 1,2-*DCB* calculated by this approximation (Figure 10a,b) was found to be close to the average diameter of the pores of CNFs obtained by the BET method (Table 3).

It is worth noting that the carbon nanofibers doped with nitrogen, CNF-Cl-N, have a greater adsorption capacity than the CNF-Cl sample. The achieved experimental maxima were 0.0051 mol·g^−1^ and 0.0037 mol·g^−1^ for CNF-Cl-N and CNF-Cl, respectively. It is assumed to be related to the larger pore volume and the more tailored pore structure in the case of the CNF-Cl-N sample (Table 3). At the same time, a high level of pore filling (F), 84% for CNF-Cl-N and 76% for CNF-Cl, distinguishes both the nanomaterials. Moreover, the synthesized CNFs have higher adsorption capacities in relation to 1,2-*DCB* if compared with other carbon materials described in the scientific literature. For example, a value of 0.00019 mol·g^−1^ was achieved for graphite nanosheets prepared by the wet ball milling of expanded graphite [38], a value of 0.00027 mol·g^−1^ for carbon nanotubes prepared by the catalytic pyrolysis of a propylene–hydrogen mixture [41], a value of 0.00179 mol·g^−1^ for the carbon material Sibunit [21], a value of 0.0019 mol·g^−1^ for industrial multiwalled carbon nanotubes NC7000 [61], and values of 0.00147 and 0.0028 mol·g^−1^ for commercially available activated carbons AG-2000 [21] and AG-5 [61], respectively.

In addition, the experimental adsorption isotherms of 1,2-*DCB* showed that the deposition of 1.5 wt% palladium has very slight effect on the adsorption capacity of CNF material (Figure 9). However, the average diameter of the pores filled by 1,2-*DCB* estimated using the Dubinin–Astakhov model is increased in this case (Figure 10). It appears that Pd nanoparticles (Figure 8) formed during the catalyst preparation partially block pores of a small diameter, and the adsorption of 1,2-*DCB* occurs in larger mesopores (Figure 10).

Thus, the adsorption of 1,2-*DCB* on the CNF-Cl-N sample occurs in a mode of the volume filling of pores, which are most likely located between the adjacent segments in the structure of carbon nanofibers.

### 3.3. Adsorptive-Catalytic Cycle of the Purification of 1,2-DCB Aqueous Emulsions

**Figure 11:**
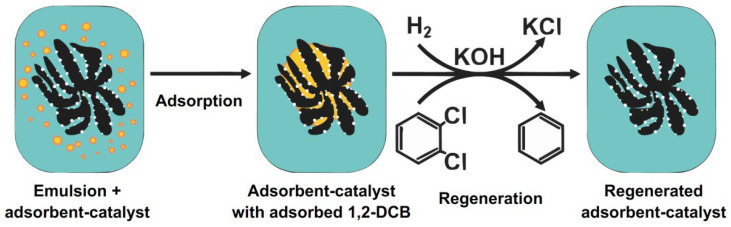
Scheme of the regeneration of adsorbent-catalysts by hydrodechlorination.

When a large amount of 1,2-*DCB* enters water, a stable emulsion is formed due to a low solubility of 1,2-*DCB* in water (0.986 mM at 25 °C). Thus, the development of the adsorption method for the purification of aqueous emulsions from 1,2-*DCB* with subsequent regeneration of adsorbent is of practical importance. In this stage of research, the efficiency of using the Pd-containing adsorbent-catalysts based on the synthesized CNF was studied for the purification of 1,2-*DCB* aqueous emulsions in the adsorptive-catalytic cycle. The process was carried out in two stages: (I) adsorption of 1,2-*DCB* on the adsorbent-catalyst at 25 °C; (II) regeneration of the adsorbent-catalyst by the liquid-phase hydrodechlorination of 1,2-*DCB* with molecular hydrogen [21] in the presence of KOH to remove formed HCl from the reaction medium (Figure 11).

The adsorption of 1,2-*DCB* was performed at a ratio of the weight of carbon nanomaterial to the volume of emulsion equal to 5 g_Pd/CNF_·L^−1^. As a result, the adsorbate concentration was reduced from 13 mM to 1.2 mM for Pd/CNF-Cl and to 1.1 mM for Pd/CNF-Cl-N. The achieved adsorption capacities were also quite similar, being 2.36 and 2.38 mmol·g^−1^ for Pd/CNF-Cl and Pd/CNF-Cl-N samples, respectively. Such a small difference is thought to be due to similar textural properties of the applied carbon materials.

After the adsorption of 1,2-*DCB* from an aqueous emulsion, the Pd/CNF adsorbent-catalysts were placed in 11 mL of 2-propanol (organic phase of the reaction medium) for the regeneration by liquid-phase hydrodechlorination with molecular hydrogen at 25 °C. It was shown that 1,2-*DCB* releases from the pore space of the adsorbent-catalyst by 2-propanol during the first minute (1,2-*DCB* concentration is 107 ± 1 mM) and then undergoes complete dechlorination. It should be noted that 2-propanol competes with 1,2-*DCB* for adsorption sites on the surface of carbon material [48].

**Figure 12:**
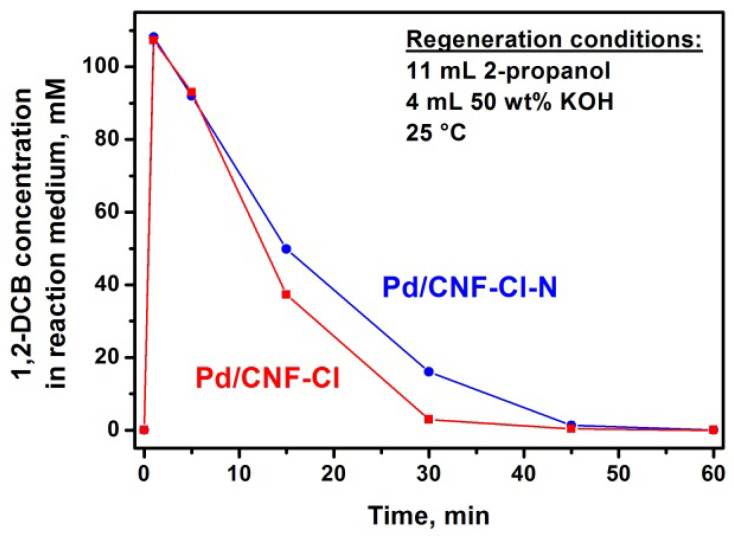
1,2-*DCB* concentration changes in the reaction medium during the regeneration of Pd/CNF-Cl and Pd/CNF-Cl-N adsorbent-catalysts (25 °C, P(H_2_) = 1 atm).

According to the data shown in Figure 12, the regeneration of Pd/CNF-Cl occurs faster than that of the nitrogen-containing Pd/CNF-Cl-N sample. The presence of additional N-containing groups (Table 1) appears to have a negative impact on the regeneration of the adsorbent-catalyst. Meanwhile, it should be noted that the regeneration time was less than 1 h for both of the studied samples.

**Figure 13:**
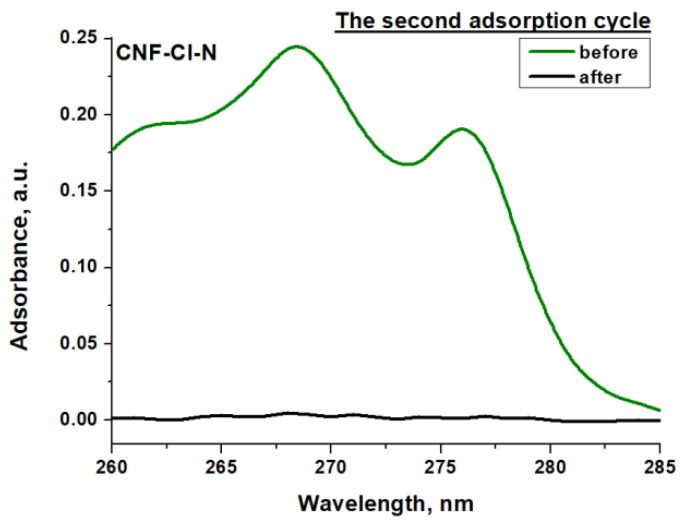
UV-vis spectrum of 1,2-*DCB* in aqueous solution before and after the second adsorption cycle for Pd/CNF-Cl-N adsorbent-catalyst at 25 °C.

After regeneration, the adsorbent-catalysts were used for post-treatment of the solution with the remaining 1,2-*DCB* after the first adsorption procedure. According to UV-vis spectroscopy data (Figure 13), the repeated use of regenerated adsorbent-catalysts made it possible to achieve almost complete removal of 1,2-*DCB* from the aqueous medium. However, its amount was very small, and during the subsequent regeneration, it was possible to identify only trace amounts of benzene after 15 min.

Thereby, the carbon nanomaterials synthesized via combined catalytic pyrolysis of the Cl- and N-containing organic wastes could be also considered as promising adsorbents for water purification from chloroaromatic compounds. Their subsequent regeneration by means of the hydrodechlorination reaction enables one to obtain chemically valuable products that can be returned to the production cycle. Thus, this approach represents another step toward the rational utilization of hydrocarbon resources.

## 4. Conclusions

In this paper, carbon nanofibers were prepared by the catalytic decomposition of trichloroethylene (or a mixture of trichloroethylene with acetonitrile) over Ni-catalyst. In both cases, CNF showed a unique segmental structure of densely and loosely packed Cl-containing graphite layers, as well as high specific surface areas (295 ± 3 m^2^·g^−1^) and porosity. According to XPS data, nitrogen atoms were embedded on the CNF surface as N≡C and C-(NC)-C (graphitic nitrogen in aromatics) groups. Furthermore, there was an increase in chlorine content and a decrease in oxygen concentration. In addition, the N-containing CNF sample was characterized by a more tailored pore structure. It had a 20% higher total pore volume (0.69 cm^3^·g^−1^ vs. 0.55 cm^3^·g^−1^) due to the greater contribution of mesopores.

The synthesized Pd-containing adsorbent-catalysts based on the produced CNF samples were explored in the adsorption of 1,2-*DCB* from aqueous medium. It was found that carbon materials efficiently adsorb 1,2-*DCB* from aqueous solutions, and the adsorption process proceeds via the pore volume filling mechanism. The presence of palladium nanoparticles was found to have negligible effect on the adsorption capacity of CNF. The increased pore volume of N-containing CNF results in a higher adsorption degree of 1,2-*DCB* from its aqueous solution. However, when CNF samples were used for purification of emulsions with high concentrations of 1,2-*DCB*, the adsorption capacities achieved were quite similar. Further regeneration of the Pd/CNF adsorbent-catalysts by reductive liquid-phase hydrodechlorination was completed in 1 h with 100% yield. The use of regenerated adsorbent-catalysts in the second adsorption cycle made it possible to achieve almost complete removal of 1,2-*DCB* from the aqueous emulsion.

Thus, in this work, a complex approach to the disposal of chlorinated organic multi-component wastes was suggested. In the first stage, carbon materials were produced by utilizing a mixture of chlorine- and nitrogen-containing organic pollutants. The produced CNF materials were then effectively used for water purification from chloroaromatic compounds with further single-stage liquid-phase regeneration by hydrodechlorination at low temperature. The resulting products can be recycled as valuable chemicals.

## Figures and Tables

**Table 1:** XPS data of the surface composition of the CNF-Cl and CNF-Cl-N samples.

CNF Sample	Content, wt%		
	N	O	Cl
CNF-Cl	0.07	2.65	0.09
CNF-Cl-N	0.39	1.47	0.17

**Table 2:** Results of the quantitative XPS data analysis.

CNF Sample	Element	BE, eV	Atomic Ratio to Carbon	State Portion	Total Atomic Ratio to Carbon
CNF-Cl	O1s	532.4	0.0049	0.18	0.027
		533.8	0.022	0.82	
	N1s	400.8	0.00057	0.75	0.00077
		402.7	0.00020	0.25	
CNF-Cl-N	O1s	531.0	0.00088	0.059	0.015
		532.1	0.0039	0.26	
		533.6	0.010	0.68	
	N1s	398.5	0.0013	0.33	0.0040
		401.2	0.0027	0.67	

**Table 3:** Textural characteristics of the CNF-Cl and CNF-Cl-N samples. BET analysis data.

Sample	*SSA*, m^2^·g^−1^	*V_∑pore_*, cm^3^·g^−1^	*V_micropo_*_re_, cm^3^·g^−1^	$\bar{d}$, nm
CNF-Cl	297	0.55	0.017	7.4
CNF-Cl-N	292	0.69	0.019	9.4

**Table 4:** Values of parameters calculated from the Langmuir and Dubinin–Astakhov adsorption isotherms for 1,2-*DCB* on the CNF-Cl, Pd/CNF-Cl, CNF-Cl-N, and Pd/CNF-Cl-N samples.

Isotherm	Linear Form	Parameter		Sample			
				CNF-Cl	Pd/CNF-Cl	CNF-Cl-N	Pd/CNF-Cl-N
Langmuir	$\frac{1}{A}=\frac{1}{A_{\max}}+\frac{1}{K_{L}A_{\max}}\times \frac{1}{C_{eq}}$ $\omega =\frac{SSA}{A_{\max}N_{A}}$	$A_{\max}$ (mol·g^−1^)		0.0022	0.0034	0.0027	0.0030
		ω (nm^2^)		0.22	0.14	0.18	0.16
		R^2^		0.710	0.965	0.714	0.840
Dubinin–Astakhov	$\ln A=\ln A_{\max}-{\left[\frac{RT}{E_{ads}^{eff}}\times \ln\frac{C_{\max}}{C_{eq}}\right]}^{n}$ $d=2\times \frac{K_{C_{6}H_{6}}\times \beta }{E_{ads}^{eff}}$	*n* = 1	$A_{\max}$ (mol·g^−1^)	0.0038	0.0045	0.0048	0.0053
			$W_{0}$ (cm^3^·g^−1^)	0.43	0.51	0.54	0.60
			$E_{ads}^{eff}$ (kJ·mol^−1^) *	5.0	4.3	6.4	4.7
			d (nm) *	6.2	7.1	8.5	6.6
			R^2^	0.924	0.969	0.950	0.935
		*n* = 2	$A_{\max}$ (mol·g^−1^)	0.0025	0.0030	0.0033	0.0035
			$W_{0}$ (cm^3^·g^−1^)	0.29	0.34	0.38	0.39
			$E_{ads}^{eff}$ (kJ·mol^−1^) *	7.7	6.5	9.6	7.5
			d (nm) *	4.0	4.7	3.2	4.1
			R^2^	0.733	0.927	0.765	0.783

* The average value.

## Data Availability

Data is contained within the article.

## Supplementary Materials

The following supporting information can be downloaded at: https://www.mdpi.com/article/10.3390/ma15238414/s1, Table S1: The quantification of XPS peaks for the sample CNF-Cl; Table S2: The quantification of XPS peaks for the sample CNF-Cl-N.
