# Supplementary material for: Synthesis of Chlorine- and Nitrogen-Containing Carbon Nanofibers for Water Purification from Chloroaromatic Compounds
Source: Materials (Basel). 2022 Nov 25;15(23):8414. doi: 10.3390/ma15238414 (PMC9741473; doi:10.3390/ma15238414)
Supplement: Supplementary file 1 [file materials-15-08414-s001.zip › materials-2050351-supplementary.pdf]

SUPPORTING INFORMATION

Synthesis of Chlorine- and Nitrogen-Containing Carbon Nanofibers for Water Purification from Chloroaromatic Com-pounds

Table S1. The quantification of XPS peaks for the sample CNF-Cl.

Element	*ASF*	*BE, eV*	*FWMH*	*AREA*	*SUM*	*X/C*	*X(sum)/C*	*X at. %*	*State portions*
C1s	0.0260	284.5	1.7	114196	199582				
		285.8	3.6	72638					
		290.4	3.7	12748					
O1s	0.0537	532.4	2.5	2015	11252	0.0049	0.027	2.65	0.18
		533.8	3.6	9238		0.022			0.82
N1s	0.0393	400.8	1.3	173	232	0.00057	0.00077	0.075	0.75
		402.7	2.2	59		0.00020			0.25
Cl2p	0.0706	200.2	1.4	331	497	0.00061	0.00092	0.089	
				166		0.00031			

Table S2. The quantification of XPS peaks for the sample CNF-Cl-N.

Element	*ASF*	*BE, eV*	*FWMH*	*AREA*	*SUM*	*X/C*	*X(sum)/C*	*X at. %*	*State portions*
C1s	0.0260	284.5	1.8	129813	177737				
		286.1	2.8	30249					
		290.2	4.7	17676					
O1s	0.0537	531.0	3.3	325	5511	0.00088	0.015	1.47	0.059
		532.1	3.7	1434		0.0039			0.26
		533.6	3.3	3752		0.010			0.68
N1s	0.0393	398.5	2.2	350	1076	0.0013	0.0040	0.39	0.33
		401.2	2.4	726		0.0027			0.67
Cl2p	0.0706	200.9	1.6	547	821	0.0011	0.0017	0.17	
				274		0.00057			
